# Abscopal effect triggered by radiation sequential mono-immunotherapy resulted in a complete remission of PMMR sigmoid colon cancer

**DOI:** 10.3389/fimmu.2023.1139527

**Published:** 2023-03-20

**Authors:** Pu Zhou, Yan Wang, Si Qin, Yan Han, Yumeng Yang, Liang Zhao, Quan Zhou, Wenlei Zhuo

**Affiliations:** ^1^ Department of Oncology, People’s Hospital of Shapingba District, Chongqing, China; ^2^ Department of Oncology, Second Affiliated Hospital of Army Military Medical University, Chongqing, China; ^3^ Department of Oncology, People’s Hospital of Chongqing Hechuan, Chongqing, China

**Keywords:** abscopal effect, immunotherapy, radiation, PMMR, MRCC

## Abstract

**Background:**

Radiation therapy combined with immune checkpoint inhibitors (ICIs) has recently turned into an appealing and promising approach to enhance the anti-tumor immunity and efficacy of immunological drugs in many tumors. Abscopal effect induced by radiation is a phenomenon that often leads to an efficient immunity response. In this study, we investigated whether the combination of the immunogenic effects derived from radiotherapy sequential ICIs-based therapy could increase the incidence of abscopal effects, and improve the survival rates.

**Case presentation:**

We described a clinical case regarding a 35-year-old male patient who was admitted to our hospital with a diagnosis of adenocarcinoma of the sigmoid colon and synchronous multiple liver metastases following a surgical resection. The molecular pathological examination showed immune-desert phenotype and proficient mismatch repair (pMMR). The patient was treated with adjuvant chemotherapy after surgery, however, after 7 months, multiple metastasis in the pelvic lymph nodes were diagnosed. Unfortunately, the tumor progressed despite multiple cycles of chemotherapy combined with cetuximab or bevacizumab. Within the follow-up treatment, the patient was administered with only 50Gy/25F of radiation dose to treat the anastomotic lesions. Subsequently, mono-sindilizumab was used as systemic therapy, leading to a rapid reduction of all pelvic lesions and complete clinical remission. So far, the patient survived for more than 20 months under continuous mono-sindilizumab treatment and is still in complete remission.

**Conclusion:**

A localized radiotherapy combined with a sindilizumab-based systemic therapy may overcome the immune resistance of pMMR metastatic colorectal cancer (mCRC), thus obtaining greater efficacy of the therapy. Its mechanism may be related to the abscopal effect obtained by the synergistic use of radiation and sindilizumab, which should be further investigated in the future.

## Introduction

ICIs have been extensively used to treat many cancers, leading to significant improvements in the survival rates for some patients (1). Patients with pMMR (about 95% of mCRC) showed a lower tumor mutational burden (TMB), and TME, with absent or inactive cytotoxic T lymphocytes (CTLs), and reduced expression of checkpoint proteins, with subsequent impairment of the response to ICIs-based monotherapy (2, 3). It is well known that radiotherapy (RT) stimulates a “bystander effect” inducing the antigen presentation, CTLs recruitment, and positive change of the tumor microenvironment (4, 5). Despite the first recorded clinical case of abscopal effect in 1908, this is still rarely described (2). The stimulated immunity following a RT cycle should increase the frequency of the abscopal effect. To this regard, integrated strategies including ICIs combined with locoregional radiation was found to be a promising treatment strategy to enhance the anti-tumor immunity (6). However, little is still known about the underlying mechanisms and no clear guidelines regarding the relationship between abscopal effect and combination therapy are currently in place. Herein, we report a unique case in which abscopal effect was observed in a pMMR mRCC patient treated first with mono-sindilizumab therapy followed by RT.

## Case description

**Figure 1:**
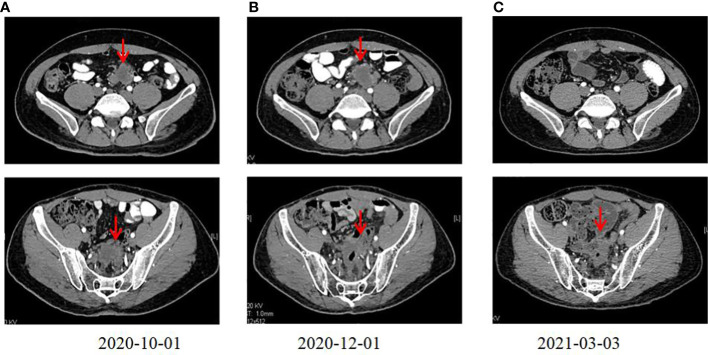
CT scanning showing the chronological response of the patient after a cycle of treatments. **(A)** CT before radiation. **(B)** CT after ten days of radiation revealing lesions. **(C)** CT revealing an abscopal effect following the sequential immunotherapy performed.

**Figure 2:**
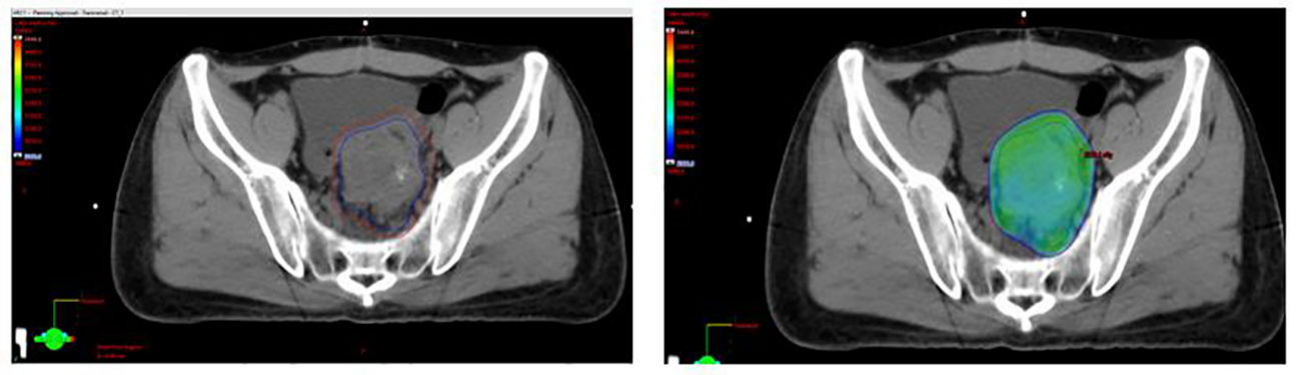
Selected axial slices representing a spatial dose-distribution using an intense and modulated radiation therapy strategy.

**Figure 3:**
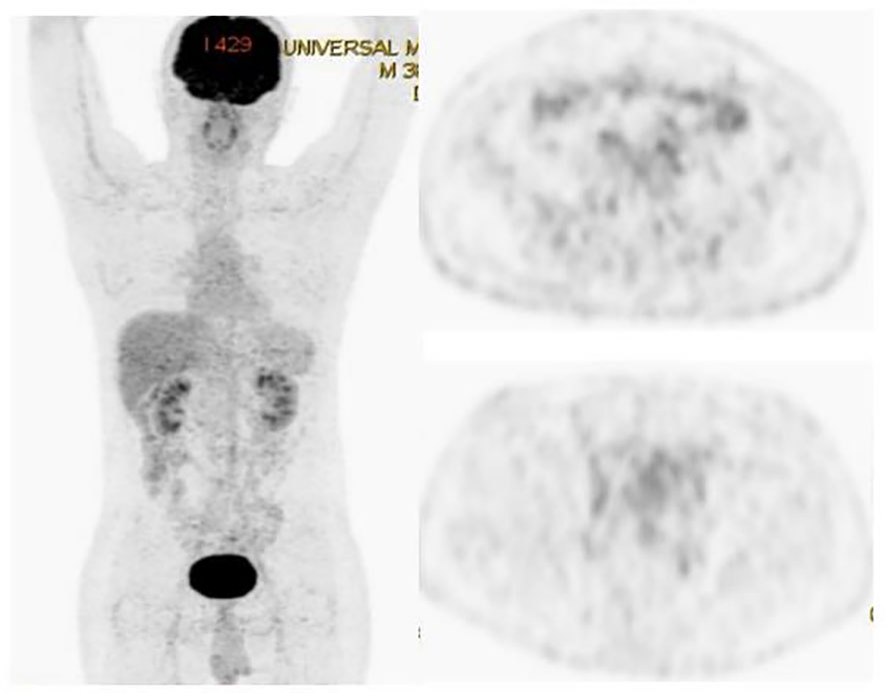
PET-CT showing the remission of all lesions following the treatment.

**Figure 4:**
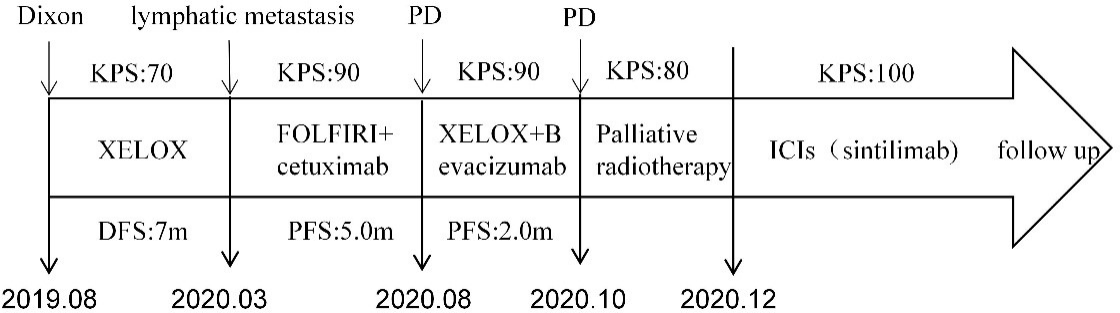
Timeline of the treatment plan following the surgery. PD, progressive disease; m, months; PFS, progression free survive; DFS, disease-free survival; KPS, Karnofsky Performance Status (KPS) refer to the Karnofsky performance status during that period.

**Figure 5:**
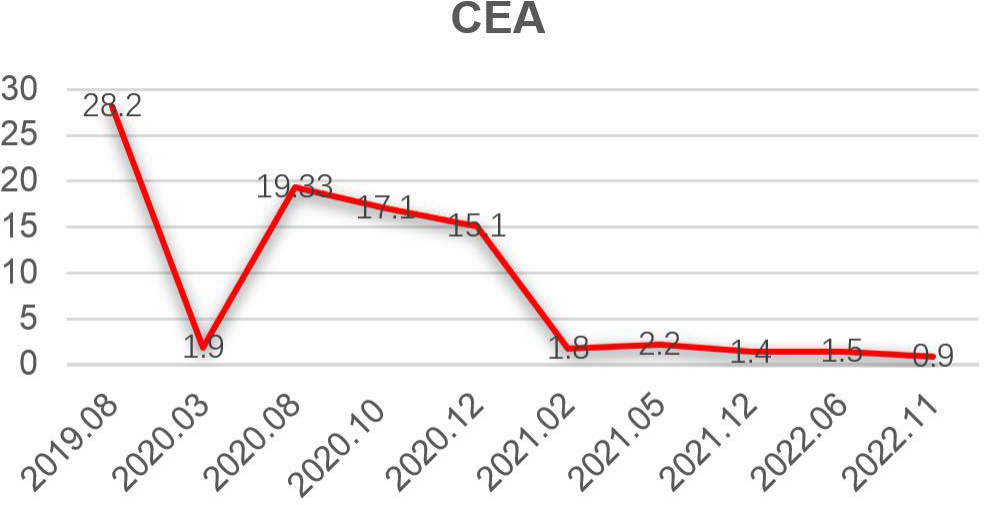
Carcinoembryonic antigen (CEA) levels during treatment.

A 35-year-old man with a diagnosis of primary sigmoid colorectal cancer was admitted to the Second Affiliated Hospital of Army Military Medical University in August 2019. Computed tomography (CT) and high-resolution magnetic resonance imaging (MRI) revealed a tumor mass in the sigmoid colorectal region accompanied by perforation and multiple liver metastases. The patient was in bad condition (karnofsky performance status (KPS) = 70) and complained of moderate abdominal pain with weight loss. He has concomitant anemia (grade 1) and a history of tobacco use for 10 years; no history of alcohol or drug abuse; no family history of colorectal cancer, polyps, inflammatory bowel disease, or other malignancies. Later, the radical resection of rectal cancer (Dixon) was performed in laparoscopy with a simultaneous resection of liver metastasis. Pathological examination confirmed a poor differentiated tubular adenocarcinoma classified as stage IV based on the TNM staging of T4N0M1 (the American Joint Committee on Cancer (AJCC) staging manual, version 8). Immunohistochemistry testing performed on a tumor biopsy sample revealed a pMMR, a negative programmed death ligand-1 (PD-L1) result, combined positive score (CPS)<1, and a Ki67 proliferative index of 80-90%. Additionally, next generation sequencing (NGS) analysis was used to demonstrate that the tumor was KRAS/BRAF/NRAS wild type. After surgery, he was treated with six cycles of the adjuvant chemotherapy drug, XELOX (oxaliplatin+capecitabine). In March 2020, metastasis in multiple pelvic lymph nodes were diagnosed, including the mesenteric (18×18 mm) and anastomotic (19×13 mm) ones. Afterwards, a treatment with the chemotherapeutic drug, FOLFIRI (5-fluorouracil+irinotecan+leucovorin), in combination with cetuximab was performed for six cycles. In August of the same year, the patient showed lower abdomen pain and the CT exam revealed a further progression of the disease up to the enlargement of two other big lymph nodes (36×42 mm; 34×21 mm). Consequently, the patient was treated with XELOX (oxaliplatin+capecitabine) combined with bevacizumab for 4 cycles. However, the patient’s lower abdominal pain persisted and the efficacy assessment test suggested a further progression in October 2020 (**Figure 1A**). An intensity modulated radiation therapy was used from October 16, 2020, to November 19, 2020. To this regard, the gross target volume (GTV) was used to determine only para-anastomotic lymph nodes, while the planning gross target volume (PGTV) was a 5-mm extension of the GTV visualized in three dimensions. A total radiation dose of 50Gy/25F was delivered to the PGTV (**Figure 2**). Surprisingly, ten days after the treatment, CT scan analysis revealed not only an enlargement of the radiotherapy lesion (40×50mm), but also of the mesenteric lymph node lesions which were not treated with RT (40×41 mm) (**Figure 1B**). On December 3, 2020, the patient received sintilimab, a PD-1 antibody (Ab) (200 mg on D1, every 3 weeks). After three months, the patient’s lower abdominal pain disappeared and serum carcinoembryonic antigen (CEA) levels returned within normal ranges with a regression of two target lesions, including the one located in the mesenteric lymph node not treated with radiation (**Figure 1C**). In November 2022 positron emission tomography-computed tomography (PET-CT) showing the remission of all lesions following the treatment (**Figure 3**). Until November 2022, the patient was continuously treated with sindilizumab and reached a progression free survive (PFS) of more than 23 months since the beginning of the immune monotherapy. The timeline of the treatment plan following the surgery and CEA levels during treatment are showed in **Figures 4**, **5**. After 2 years of sindilizumab maintenance therapy, the patient was regularly seen for follow up every 6 months without further antineoplastic treatment and generally in good clinical condition (KPS 100) without the onset of new relevant symptoms. The last CT scans were performed in February 2023, and there was still no sign of tumor progression.

## Discussion

The standard application of RT was influenced by the recent therapeutic breakthroughs in the field of cancer immunotherapy (7, 8).To this regard, RT triggers a systemic immune response, however, the impairment of the immunological functionality of different combined radiotherapy regimens remains controversial and needs to be further investigated (5). The combined immunotherapy-based approaches have already been the focus of several research studies, but several failures have been reported more in pMMR than in dMMR colorectal cancer patients (2). Therefore, it should be investigated a possible treatment combination to enhance the immunotherapy effect in pMMR mCRC patients. Our previous research found that in human tissue specimens after 1 week of RT, PD-L1 expression along with neo-mutations of tumor cell genes increased significantly in the TME (9). Radiation can cause DNA breakage and damage tumor cells, and promote the release of a large number of tumor associated antigen (TAA) fragments into the blood. Dendritic cells recognize and phagocytose these TAA, further present tumor antigen and activate cytotoxic CD8+T lymphocytes. Finally, activated tumor antigen-specific T cells are recruited to attack tumor cells outside the radiation field, resulting in abscopal effect (10–12).

Despite the abscopal effect is a rare phenomenon, it was found that RT combined with ICIs may improve the effectiveness of the antitumor immunity increasing the frequency of the abscopal effect, especially when RT is followed by ICIs treatment after a week (13). However, due to the immune tolerance of tumor cells to T cells, although there is a correlation between the distant effect and the activated immune system, it is unpredictable to observe systemic antitumor activity when using RT (14). As a result, only a few successful cases have been reported. On the other hand, it has been proved that the expression and activity of PD-1 on T cells and PD-L1 on tumor cells down-regulate immune activity through T cell apoptosis. PD-1/PD-L1 antibody can relieve the immune tolerance of tumor cells to T cells and amplify the immune response to promote anti-tumor effects. Mechanically, PD-1/PD-L1 antibody may be used to enhance the abscopal effect induced by radiotherapy (14, 15).

In this case, the lesion outside the radiotherapy area (non-radiotherapy mesenteric lymph nodes) is we want to emphasize, where the abscopal effect might occur. Meanwhile, its pathological type (pMMR) is primary resistant to PD-1 antibody, so PD-1 antibody alone will not have effect. However, when the anti-PD-1 antibody was later used as a systemic treatment, not only the lesions in the radiotherapy area, but also the lesions outside the radiotherapy area (the mesenteric lymph nodes) were significantly subsided. For the lesions in the radiotherapy area, its regression can be interpreted as a synergism of direct killing effect of radiotherapy and immune enhancement effect of pd-1 antibody. However, for the lesions outside the radiotherapy area (non-radiotherapy mesenteric lymph nodes), its regression may be caused by the abscopal effect promoted by PD-1 antibody. According to the immunological mechanism, radiation acts on tumor cells in the radiotherapy field, inducing tumor cell death and tumor associated antigen release. These tumor-related antigens activate cytotoxic T cells that target tumor cells through the mediation and presentation of dendritic cells. Then, the subsequent application of PD-1 antibody can relieve the immune tolerance of tumor cells to T cells and promote the anti-tumor effect of T cells.

In conclusion, we presented a case of pMMR mCRC with long-term clinical benefits deriving from the use of new approaches based on a simultaneous use of immunotherapy and RT, which induced the abscopal effect. We conclude that the understanding of the potential benefits of combined therapies is essential to design optimized treatment strategies targeting severe cancers, like pMMR mCRC.

## Data availability statement

The original contributions presented in the study are included in the article/supplementary material. Further inquiries can be directed to the corresponding author.

## Author contributions

WZ developed the study design. PZ, YW, and YH contributed to Patient management and follow-up. YY, LZ, and QZ performed analyze. All authors contributed to the article and approved the submitted version. 
